# SNR analysis of contrast-enhanced MR imaging for early detection of rheumatoid arthritis

**DOI:** 10.1371/journal.pone.0213082

**Published:** 2019-03-01

**Authors:** Florian T. Gassert, Felix G. Gassert, Geoffrey J. Topping, Ernst J. Rummeny, Moritz Wildgruber, Reinhard Meier, Melanie A. Kimm

**Affiliations:** 1 Department of Diagnostic and Interventional Radiology, Klinikum rechts der Isar, Technische Universitaet Muenchen, Munich, Germany; 2 Department of Nuclear Medicine, Klinikum rechts der Isar, Technische Universitaet Muenchen, Munich, Germany; 3 Translational Research Imaging Center, Department of Clinical Radiology, Universitätsklinikum Muenster, Muenster, Germany; Medical University of Vienna, AUSTRIA

## Abstract

**Objective:**

To investigate whether signal to noise (SNR) analysis of contrast-enhanced MRI gives additional benefit for early disease detection by Magnetic Resonance Imaging (MRI) of experimental rheumatoid arthritis (RA) in a small animal model.

**Methods:**

We applied contrast-enhanced MRI at 7T in DBA mice with or without collagen-induced arthritis (CIA). Clinical score, OMERACT RAMRIS analysis and analysis of signal to noise ratios (SNR) of regions of interest in RA bearing mice, methotrexate/methylprednisolone acetate treated RA and control animals were compared with respect to benefit for early diagnosis.

**Results:**

While treated RA and control animals did not show signs of RA activity in any of the above-mentioned scoring methods at any time point analyzed, RA animals revealed characteristic signs of RA in RAMRIS at the same time point when RA was detected clinically through scoring of the paws. The MR-based SNR analysis detected signs of synovitis, the earliest indication of RA, not only in late clinical stages, but also at an early stage when little or no clinical signs of RA were present in CIA animals and RAMRIS did not allow a distinct early detection.

**Conclusion:**

SNR analysis of contrast-enhanced MR imaging provides additional benefit for early arthritis detection in CIA mice.

## Introduction

With a prevalence of 0.5% to 1%, Rheumatoid Arthritis (RA) is the most common arthritis affecting the small joints of the hands and feet [1]. RA is characterized by proliferative synovitis, osteitis and cartilage destruction [2]. Untreated, it ultimately leads to the progressive destruction of articular and periarticular structures [3].

RA therapy has made great progress in recent decades, and modern therapeutic schemes aim at reducing both inflammation and structural damage. However, early diagnosis and subsequent therapy are crucial for a positive outcome [4]. Presently, a combination of scoring clinical symptoms, analyzing laboratory parameters and medical imaging is used to diagnose RA. While advanced stages of RA are easily diagnosed via clinical symptoms, early RA appears discreet and atypical [5]. MRI in particular facilitates the detection of changes of bone metabolism or articular effusion [6, 7]. Synovitis as earliest sign of RA is visible in contrast-enhanced MRI due to hypervascularization and increased edema at the inflamed site. However, early diagnosis is still challenging because changes in contrast enhancement and joint inflammation in MRI are subject to the individual interpretation of the radiologist.

Collagen-induced arthritis (CIA) in mice is a well established preclinical model for research of rheumatoid arthritis [8]. However, as with clinical diagnosis of RA, early changes in disease progression and therapy response are still difficult to identify because the accurate interpretation of MR images is dependent on the experience of the examiner. Objective analysis methods, not depending on any examiner's experience or bias, are therefore needed to assess early signs of inflammation and contrast enhancement. The SNR analysis is widely used to describe the performance of MRI systems for image evaluation and measurement of contrast agent [9]. It does not only take the average signal intensity into account, but also the background noise. Therefore it allows for comparison between various scanners and quantitative measurements which reduces examiner dependence. Hence, it is widely used for automated image analysis approaches.

In this study, we demonstrate the impact of clinical scoring with contrast-enhanced MRI and subsequent computational image analysis for the early diagnosis of joint inflammation in CIA in mice.

## Material and methods

### Animal model

Procedures involving animals and their care were conducted in conformity with national and international guidelines with approval from the local authority (Government of Upper Bavaria, 55.2-1-54-2532-179-11) and supervised by the Institutional Animal Care and Use Committee. Animals were housed in standard animal laboratories (12 h light/dark cycle) in single ventilated cages with free access to water and standard laboratory chow *ad libitum*. In total, 18 DBA/1JRj mice (8 weeks old) were obtained from Janvier Labs (France) and randomly categorized into 3 groups (n = 6 per group). One group was injected with phosphate buffered saline (PBS), representing sham animals without RA as control group (Ctrl). Two groups were subjected to arthritis-inducing collagen injections (RA and RA+Therapy). All injections and imagings were performed under isoflurane anesthesia. At the endpoint of the experiments animals were sacrificed using standard techniques. No adverse outcomes have occurred.

### Induction and treatment of CIA

12 DBA/1 mice (RA and RA+Th) were injected s.c. with 50 μg bovine type II collagen (Chondrex Inc., Redmond, USA) in Freund's complete adjuvant (Sigma Aldrich, St. Louis, USA) into the base of the tail. 14 days later, these mice were injected with 50 μg bovine type II collagen (Chondrex Inc., Redmond, USA) in Freund's incomplete adjuvant (Sigma Aldrich, St. Louis, USA). 6 control mice (Ctrl) were injected at the same time points with PBS with pH 7.2. Therapy was initiated in 6 mice (RA+Th) at day 1 after CIA induction. These animals were injected i.p. once per week with 1 mg/kg methotrexate (Sigma Aldrich, St. Louis, USA) and 4.2 mg/kg methylprednisolone acetate (Pfizer, La Jolla, USA). All animals received the opioide analgesic Buprenorphin (0.1 mg/kg) to prevent pain after CIA induction.

### Clinical scoring

Paw swelling and alterations of the extremities were monitored as previously described [10]. Briefly, each joint of the extremities and paws were measured for thickness, swelling, warmness and redness and rated on a scale of 0–4. An overall score for each mouse was calculated as the sum of its individual scores, with a maximum of 16. As all joints of the extremities were examined, the term clinical score is used instead of paw score.

### MRI

Control mice (Ctrl), animals with collagen-induced arthritis (RA) and RA mice treated with standard therapy (RA+Th) were examined before collagen induction (day -1) and twice during RA development (day 32 and day 42) with contrast-enhanced 7T MRI and clinical scoring at the same time points. The imaging time points were chosen according to the appearance of clinical symptoms (**S1 Fig**). MR images were acquired on 7T small animal MRI system (Discovery MR901, GE Healthcare, Waukesha, Wisconsin, USA and Agilent Technologies, Oxford, UK). Mice were anaesthetized with isoflurane and placed with a hind leg on a 2 cm single-channel receive coil (RAPID Biomedical, Rimpar, Germany). A 2D multislice T1 weighted spoiled gradient recalled echo sequence was used, with 5 slices of 0.25 mm slice thickness, TR 50 ms, TE 3.9 ms, flip angle 30 degrees, receive bandwidth 46.9 kHz, and 150 averages, matrix size 192x96 and field of view 15 mm x 7.5 mm. Image slices were oriented parallel to the bones of the hind leg, perpendicular to the surface of the paw. Images were acquired before, immediately after, and 20 min after tail vein injection of 100 μmol/kg body weight Gadofluorine P (invivoContrast GmbH, Berlin, Germany), an experimental contrast agent with increased affinity to extracellular matrix proteins.

### Image analysis and statistics

For MR image assessment of synovitis, the EULAR-OMERACT rheumatoid arthritis MRI reference image score (RAMRIS) was applied [7, 11]. Synovitis was scored on a scale of 0–3 in each joint, with a score of 0 representing no synovitis and scores of 1–3 representing mild, moderate, and severe arthritis by two radiologists with more than 10 years of expertise in the assessment of rheumatoid arthritis. For the signal to noise ratio (SNR) analysis, a region of interest (ROI) was drawn from the lower shank to the metatarsus, including all joints in the foot area (**S2 Fig**), and the mean signal intensity (SI) of the ROI was determined. In each image, we measured the mean SI of an area outside the animal, but next to the paw, and set it as a reference of background noise. SNR were calculated as the ratio of average signal intensity of the foot area to the standard deviation of the background [9]. The difference between SNR of the pre injection scan (pre) and the post-injection scan (post images were taken immediately after injection of contrast agent) was calculated for each mouse at every imaging time point. Images were analyzed using OSIRIX software (Pixmeo, Bernex, Switzerland).

For assessing the power of each scoring method (clinical score, RAMRIS, SNR) to detect early signs of RA, we compared the values on day 32 of the RA group by normalizing each score to percentage values. From each scoring method, the maximum value on day 42 was set to 100% and the minimum value on day -1 (baseline) was set to 0%. For calculating the percentage values for each score on day 32, the baseline values were subtracted from day 32 values and the result was given as a percentage of the difference between maximum value (day 42, 100%) and minimum (baseline) value (day -1, 0%).

A one-way analysis of variance (ANOVA) with subsequent Bonferroni post-test for multiple comparisons was applied for the SNR analysis and the comparison of the relevant scores.

Data was analyzed using GraphPad Prism version 6.0h (GraphPad Software, San Diego, USA). Data sets with n≥ 5 were tested for outliers with the Grubbs outlier test. Outliers were not included in statistical analysis. A p-value of 0.05 was considered significant and a p-value of 0.001 was considered highly significant. The Intra-Class-Correlation (ICC) for all days and time points was calculated.

### Additional histological validation

Extremities were fixed post mortem in 4% neutral buffered Formalin, decalcified in Osteosoft (Merck, Darmstadt, Germany) according to the manufacturer's instructions, and embedded in paraffin. 4 μm thick sagittal sections were stained with Movat Pentachrome (Morphisto, Frankfurt am Main, Germany) according to standard protocols. Stained sections were examined for arthritis using a Zeiss AxioImager II microscope and Zeiss imaging software AxioVision (Zeiss, Germany).

## Results

### MRI analysis of synovitis in CIA mice

**Fig 1:**
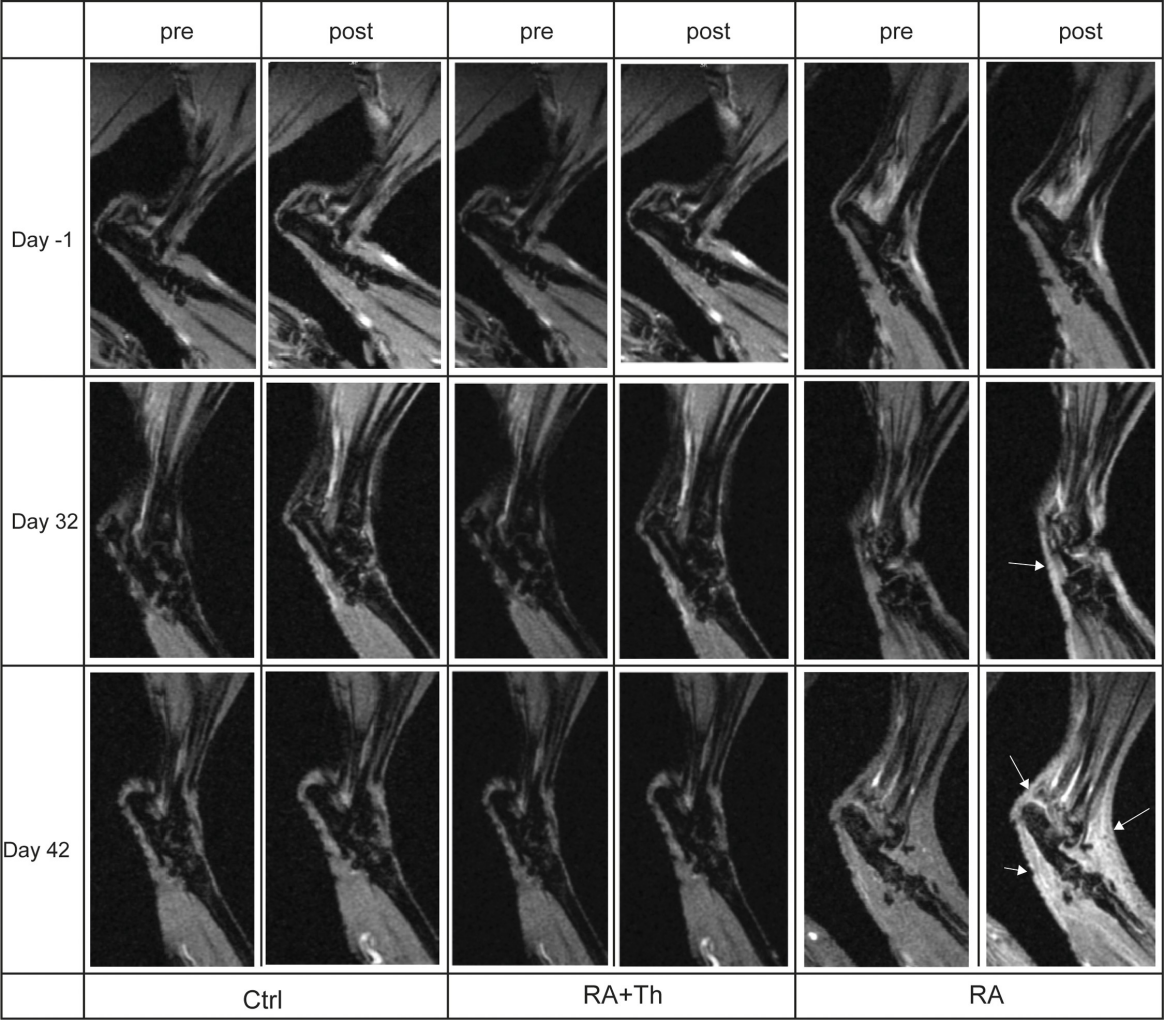
Arthritis detection in MRI. Pre injection scan images (pre) and immediate post injection scan images (post) of representative animals are shown. No signs of arthritis are found in Ctrl and RA+Th animals at any time point. First signs of synovitis are detectable in the RA animal on day 32 (arrow). On day 42 synovitis is clearly visible (arrows). Ctrl (Control), RA (animals with arthritis induction), RA+Th (animals with arthritis induction and therapy).

The MR images of one representative animal per group (Ctrl, RA, RA+Th) before (pre) and after (post) injection of Gadofluorine P are highlighted in **Fig 1**.

For each animal and time point, MR images were analyzed with respect to synovitis using the OMERACT RAMRI score. Scans of all animals on day -1 (baseline) did not show any signs of synovitis in any of the groups (**Fig 1**); no swelling or edema was present, the joint cavities were of normal size, the bone tissue border was distinct and no increased uptake of contrast agent was visible in any group. No changes of these characteristics could be observed in the Ctrl group in the following scans, resulting in RAMRI score 0 for all time points (**Table 1**).

**Table 1:** Average rheumatoid arthritis MRI reference image score (RAMRIS).

	day -1	day 32	day 42
**Control**	0	0	0
**RA+Th**	0	0	0
**RA**	0	1.3	2.3

All results are expressed as mean. Ctrl (Control), RA (animals with arthritis induction), RA+Th (animals with arthritis induction and therapy).

The same was observed for animals of the RA+Th group. Again, RAMRI score was 0 at all time points analyzed (**Table 1**). While the RAMRI score for the RA group was 0 on day -1, it increased to an average of 1.3 (equaling 57% of the reachable score) on day 32 and an average of 2.3 on day 42 (equaling 100% of the reachable score)(**Table 1**). The interobserver agreement between two readers for the RAMRI score was high, with an ICC of 0.92. In the RA group, synovial fluid collection was observed in all animals as slight swelling and increased uptake of contrast agent on day 32 by MRI (**Fig 1**, right column, middle row, arrow). However, joint cavities were still of normal size and the bone tissue borders were distinct. Whereas only 5 out of 6 animals were identified with first signs of arthritis by clinical scoring (83%), all animals (6/6) showed clinical signs in the RAMRIS analysis. On day 42, all animals of the RA group showed clinical signs and also a strongly increased uptake of contrast agent as a sign of strong synovial edema and swelling (eg. **Fig 1**, right column, bottom row, arrows) was detected in all animals with RAMRIS. The contrast agent uptake increased all over the inflamed joints, but especially in joint cavities that had broadened due to synovitis. Furthermore, the circumferences of the extremities had increased due to edema (**Fig 1**, right column, bottom row) and the bone tissue borders became blurred.

Animals of all groups were histologically analyzed after sacrifice following the final scan on day 42 (**S3 Fig**). No signs of inflammation or bone changes were detectable in the Ctrl (**S3A Fig**) and the RA+Th group (**S3B Fig**), validating the results of the MR image analysis. Only animals of the RA group (**S3C Fig**) showed all characteristic signs of synovitis and bone erosion.

### SNR analysis of MR images for RA detection

Signal to noise ratio (SNR) analysis was applied to pre and post MR images of all groups at all time points. Then, the differences of SNR before and after the injection of contrast agent was calculated (SNR difference pre to post) (**Table 2**).

**Table 2:** Average SNR value difference between pre and post injection scan images per group and imaging time point.

	day -1	day 32	day 42
**Control**	0.82 (±1.49)	0.41 (±0.89)	0.91 (±1.42)
**RA+Th**	-0.65 (±3.44)	0.99 (±3.52)	0.91 (±2.92)
**RA**	0.85 (±0.98)	6.32 (±1.09)	6.94 (±2.53)

Mean values and standard deviations (in braces). Ctrl (Control), RA (animals with arthritis induction), RA+Th (animals with arthritis induction and therapy).

**Fig 2:**
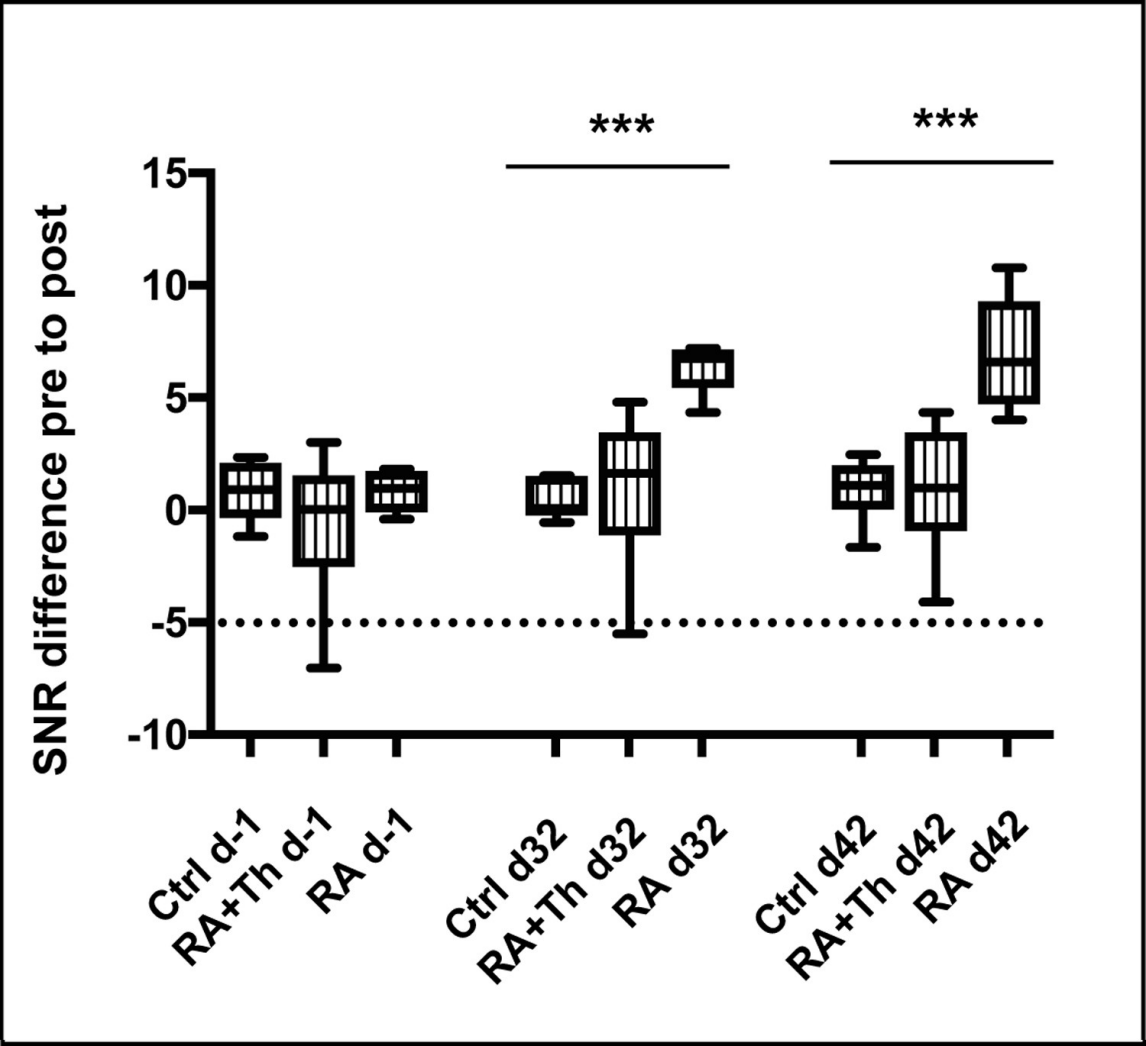
Comparison of the difference of SNR of pre to post scans. Differences in signal enhancement after contrast agent administration were analyzed on day -1, 32 and 42 and compared for Ctrl (= Control), RA (= Animals with RA induction) and RA+Th (= Animals with RA induction and therapy) group using difference in signal to noise ratio (SNR) before (pre) and after (post) injection. Values are given as boxplots (median; whiskers: min to max). Statistically significant differences between groups were depicted as: ***: p≤0.001.

The SNR analysis revealed no statistically significant difference between all three groups at baseline (p>0.05) (**Fig 2**), showing that there were no signs of arthritis visible in any animal.

Additionally, no significant difference was observed between the RA+Th and Ctrl group. This is consistent with the clinical score and the conclusion of the evaluating radiologists. On day 42, animals from the RA group presented significantly higher SNR values (p = 0.001) compared to animals from the Ctrl and RA+Th groups (**Fig 2**). At this time point, clinical signs of arthritis were also detectable in the RA group from the paw score and RAMRIS. This shows that the SNR analysis is generally able to detect synovitis as early sign of arthritis in CIA mice.

The result of the SNR analysis of the RA group at day 32 are again in agreement with the results from the two other assessed scoring methods (clinical score and RAMRIS) and show the power of the SNR analysis. Only small changes in the clinical appearance were detectable in the RA group on day 32. The examination of the MR images by two radiologists also revealed first signs of synovitis. SNR analysis resulted in significantly higher values of the RA group compared to the control group (p = 0.0006) (**Fig 2**). At all three time points, the RA+Th group did not differ significantly from the control group (day -1: p = 0,54, day 32 and day 42: p>0.9999) (**Fig 2**).

### Comparison of relevant scores for RA detection

**Fig 3:**
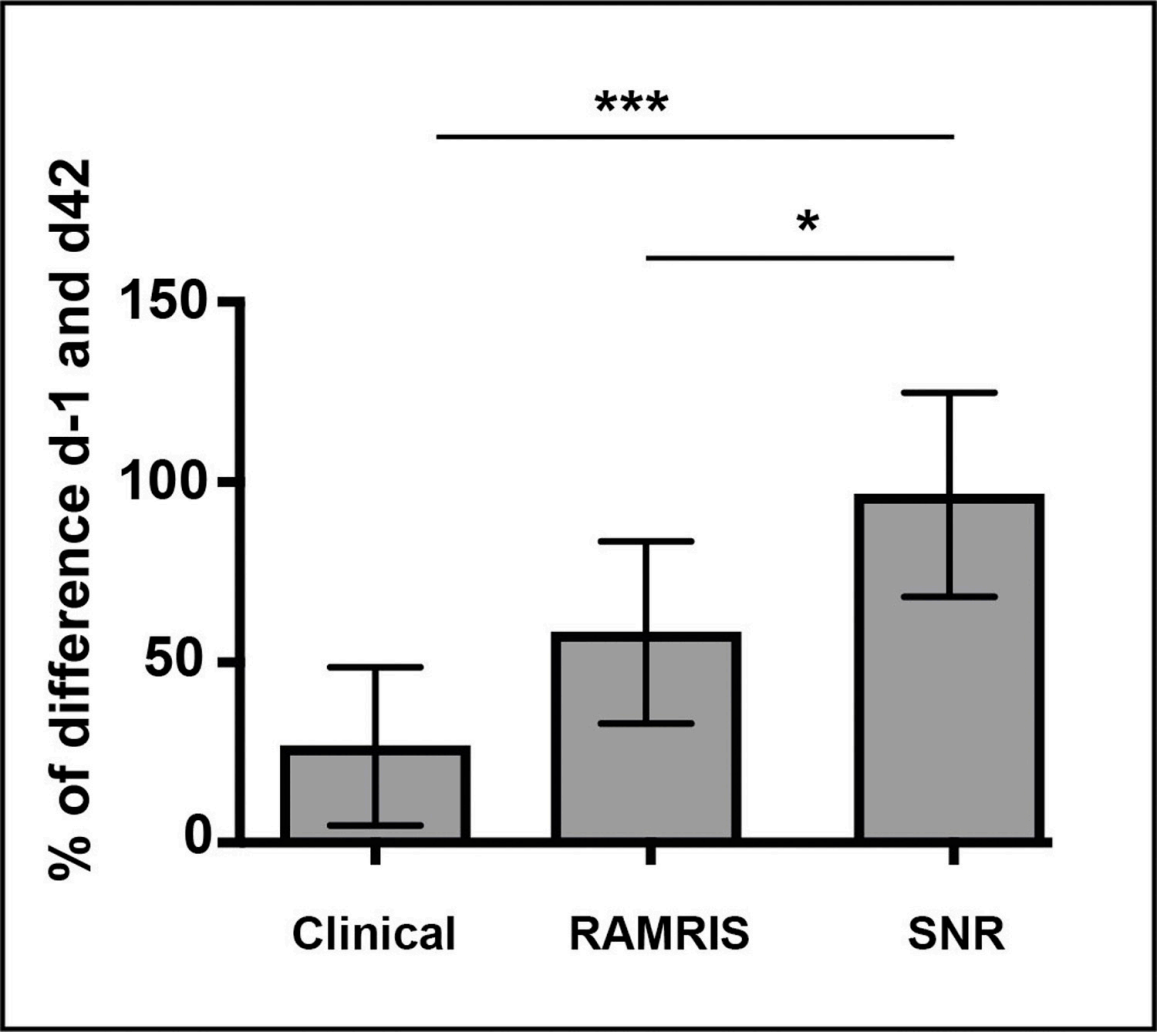
Comparison of different scorings. Relative value of clinical scoring, RAMRIS and SNR analysis on day 32 of the RA group were compared with each other (d32—d-1) / (d42—d-1). Values are given as mean ± SD. *: p<0.05, ***: p<0.001.

In a next step, we investigated the relative power of each scoring method within the RA group (as described in the method section). Normalizing the values was required in order to compare the different scoring methods which had different scales. The baseline values for the SNR were the respective values on day -1. The value of each assessment on day 42 was used as a standard, and the value of each mean on day 32 was given as a percentage of that standard and graphically represented in **Fig 3**.

The percentage value from each scoring method reveals the potential of each score to detect arthritis at an early time point. On day 32, the clinical score had reached only 26.9% (SD±17.5%) of its maximum. Conversely, all values acquired with contrast-enhanced MRI showed a much higher percentage of their maximum on day 32: RAMRIS reached 53.3% (SD±25.3%), and SNR reached 96,5% (SD±28.3%) (**Fig 3**). The percentage values of each mean indicate the severity of RA on day 32 compared to the fully developed RA on day 42. This was highest for SNR analysis and lowest for the clinical paw scoring. A SNR value of 96,5% (SD±28.3%) on day 32 indicates that the detected severity of RA on day 32 was slightly higher than that on day 42.

This shows that the SNR assessment of MR images of the RA group on day 32 showed higher potential for early RA detection compared to the state of the art scoring methods RAMRIS (p = 0.04) and clinical paw score (0.0005).

## Discussion

Clinical scoring and imaging are currently the standard methods for quantifying disease severity in CIA. Although a clinical scoring system for CIA exists [9], it is time consuming and dependent on the examiner’s experience. Both in clinics and especially in research, more accurate and less examiner-dependent techniques, such as automated image analysis, are of great interest for more-readily quantifying and comparing disease severity.

MRI has gained increasing importance in the diagnosis and quantification of RA and has been reported as a sensitive diagnostic tool in doubtful clinical scenarios as well as a specific predictor of treatment response in RA [12–14]. With suitable contrast agents, pre- and post-contrast MRI is highly sensitive in detecting local inflammation in the form of synovitis [15, 16]. Furthermore, MRI has proven to be able to detect not only clinical evident but also subclinical inflammation [17]. However, it is costly and time-consuming, and the analysis of acquired images should therefore be as precise as possible. The signal-to-noise-ratio is frequently used for image evaluation and measurement of contrast enhancement and can be used as an objective quality measure for biomedical images [9, 18]. Being a quantitative approach, the SNR is of high interest for automatic image analysis approaches [19]. However, SNR has not yet been used for facilitating an examiner-independent analysis of MR images of CIA mice. We show that the SNR analysis of contrast-enhanced MR images in mice provides high impact and benefit and allows unbiased comparison of regions of interest.

Especially, the early detection of synovitis in mice with arthritis is challenging, as details are small and subtle and changes difficult to identify. Kraben et al. examined 179 patients with early arthritis using a 1.5T MRI of metacarpophalangeal (MCP) joints, wrist and metatarsophalangeal (MTP) joints. Similar to our study, they describe that MRI could detect inflammation in 54–64% of joints [20]. Based on the signal-to-noise ratio together with case-sensitive statistics, we were able to detect RA in MRI, unbiased by a radiologist's experience. If combined with image analysis software that could automatically detect the ankle region and subsequently perform the SNR analysis, this process could be made fully automated, examiner-independent, less time consuming and more reliable. In this study, however, we aimed at proofing the concept of using SNR analysis for early detection of RA and there are probably a variety of parameters and hardware that could be used. The important point is the usage of a sequence sensitive to the contrast accumulation. Further studies are needed to set it up for a broader usage. For this aim, different scanners and settings will be needed to fully standardize this approach. Even though thresholds for RA detection by SNR change would be setup-dependent, it should be still possible to automate it once appropriate thresholds are determined for a given setup or model.

Clinical signs are often very subtle, and judging the response to novel therapeutics can be critically impeded. Furthermore, comparing different studies is, as yet, not possible, because the unbiased examiner-independent analysis of MR images of RA in mice is not yet common. Results from preclinical studies in particular are frequently used to decide if a new drug will proceed to clinical trials. It is therefore critical that studies are analyzed in as much detail as possible and in a manner comparable to other studies. Further preclinical studies with more animals are needed to demonstrate the utility of contrast enhanced MRI SNR ratio to detect inflammation in a large range of experimental set ups.

One limitation of our study is the small anatomic size of the mouse ankle with a diameter of 3–4 mm. Due to this, it was not possible to draw ROIs only around the synovia of the joint and we therefore had to include surrounding tissues. Thus, the SNR analysis was not specific for the synovia only. But as both control groups enclosed in our study did not show any changes in signal intensity or the SNR analysis, we can rule out that the presence of bone and soft tissue in the ROI leads to a false positive results in regard to synovitis. Another limitation is the necessity of drawing the ROIs manually and therefore the ROI is dependent on the examiner experience. In addition, SNR analysis of MRI images is not specific for RA but detects increased uptake of contrast agent due to inflammation in general which can also be found in other types of arthritis. As this is a problem for all examiners using CIA mice as preclinical model for RA, we aimed to find a tool to better compare the results of different studies done is this animal model. For this reason, we analysed the usage of SNR analysis and are happy to present its user friendliness and utilisability even for examiners with less experience. The inclusion of bone, synovia and soft tissue in the analysis does not present a problem as the uptake of the contrast agent is leading to a change in the SNR which finally identifies ankles with pathological changes.

In conclusion, our results show that contrast enhanced MRI in combination with SNR imaging analysis can help to detect RA in its early stages and has high potential for full automatization.

## Supporting information

S1 Fig Development of clinical symptoms of RA. Progression of clinical paw score as an average of the sum of all four paws of CIA animals of the RA group (n = 6). At day -1, day 32 and day 42 all animals were scanned with MRI (arrows). Mean values are shown for each group.
(TIF)

S2 Fig Region of interest definition. The region of interest (ROI) was drawn from the lower shank, close to the ankle joint, to the metatarsus, including all joints in the foot area. A rectangular region next to the paw was drawn to measure background noise.
(TIF)

S3 Fig Histological analysis of control and CIA-induced animals. Movat Pentachrome stained formalin fixed paraffin embedded sections of ankle joints of one representative animal per group are shown. A. Control animal with no pathological change. B. Animal with arthritis and undergone therapy with no pathological changes. C. Animal with arthritis with pathological changes. Around the joint, inflammation can be detected (asterisks) and bone erosions (arrows) are visible.
(TIF)

S4 Fig NC3Rs ARRIVE guideline checklist. (PDF)
