# Supplementary figures and images for: SNR analysis of contrast-enhanced MR imaging for early detection of rheumatoid arthritis
Source: PLoS One. 2019 Mar 1;14(3):e0213082. doi: 10.1371/journal.pone.0213082 (PMC6396898; doi:10.1371/journal.pone.0213082)

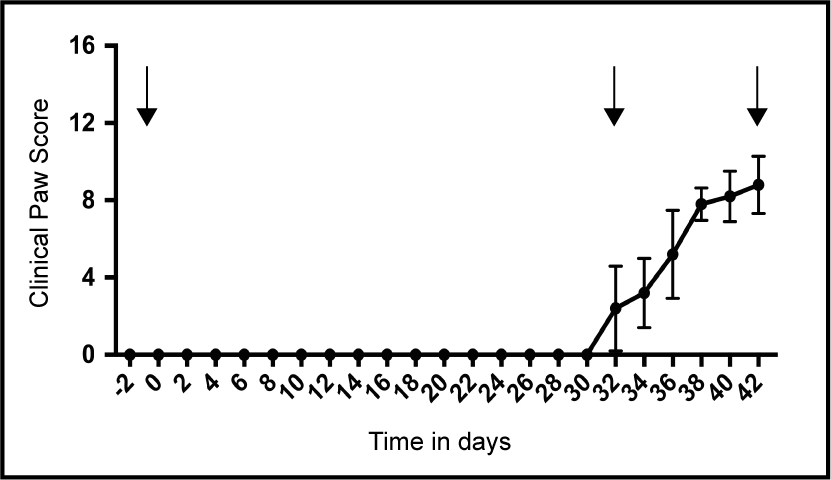

Supplement: S1 Fig — Progression of clinical paw score as an average of the sum of all four paws of CIA animals of the RA group (n = 6). At day -1, day 32 and day 42 all animals were scanned with MRI (arrows). Mean values are shown for each group. (TIF) [file pone.0213082.s001.tif]

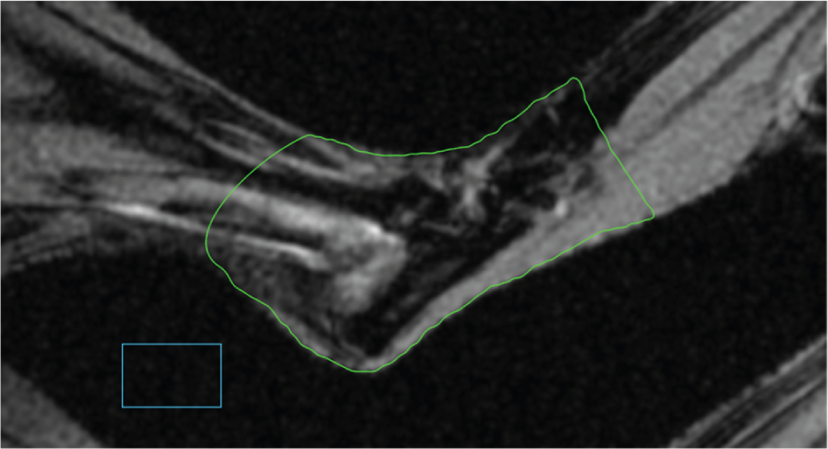

Supplement: S2 Fig — The region of interest (ROI) was drawn from the lower shank, close to the ankle joint, to the metatarsus, including all joints in the foot area. A rectangular region next to the paw was drawn to measure background noise. (TIF) [file pone.0213082.s002.tif]

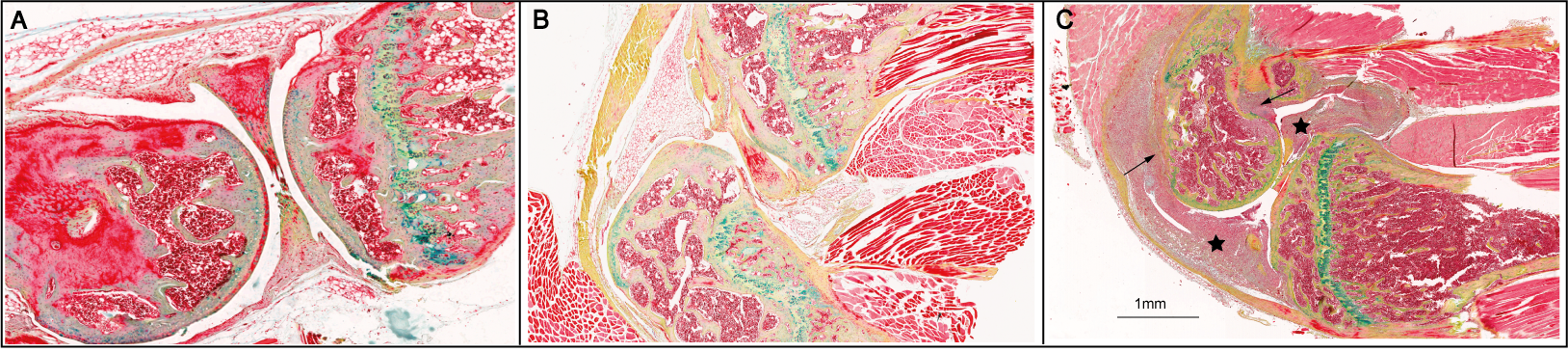

Supplement: S3 Fig — Movat Pentachrome stained formalin fixed paraffin embedded sections of ankle joints of one representative animal per group are shown. A. Control animal with no pathological change. B. Animal with arthritis and undergone therapy with no pathological changes. C. Animal with arthritis with pathological changes. Around the joint, inflammation can be detected (asterisks) and bone erosions (arrows) are visible. (TIF) [file pone.0213082.s003.tif]
